# Central role for protein kinase C in oxytocin and epidermal growth factor stimulated cyclooxygenase 2 expression in human myometrial cells

**DOI:** 10.1186/1756-0500-7-357

**Published:** 2014-06-10

**Authors:** Elien Wouters, Claire A Hudson, Craig A McArdle, Andrés López Bernal

**Affiliations:** 1 Current address: Hasselt University, Campus Diepenbeek, Biomedical Research Institute Agoralaan, building C, 3590 Hasselt, Diepenbeek, Belgium; 2 University of Bristol, School of Clinical Sciences (Obstetrics and Gynaecology), Dorothy Hodgkin Building, Whitson Street, Bristol BS1 3NY, UK

**Keywords:** Prostaglandin synthase, Uterus, Regulation, Intracellular pathways, Parturition

## Abstract

**Background:**

Prostaglandins are important mediators of uterine contractility and cervical ripening during labour. Cyclooxygenase-2 (COX-2), also known as prostaglandin-endoperoxide synthase 2, is a rate limiting enzyme involved in the conversion of arachidonic acid into prostaglandins at parturition. In this paper, the pathways underlying agonist-induced cyclooxygenase-2 expression in human myometrial cells were studied.

**Results:**

Myometrial cells were stimulated with different agonists: oxytocin (OXT), epidermal growth factor (EGF), interleukin-1β (IL1β), and phorbol-12-myristate-13-acetate (PMA) alone and in the presence of specific signalling pathway inhibitors. The nuclear factor kappa-light-chain-enhancer of activated B cells (NFKB) pathway was inhibited by means of the IKK-2 inhibitor TPCA-1. Signalling through extracellular signal-regulated kinases (ERK) was inhibited using the MEK1/2 inhibitor PD-184352. Bisindolylmaleimide-I was used to inhibit protein kinase C (PKC) signalling. COX-2 expression and ERK phosphorylation were measured using immunoblotting.

OXT induced COX-2 expression by activating PKC and ERK. EGF increased COX-2 expression via stimulation of PKC, ERK and NFKB. As expected, the pro-inflammatory cytokine IL1β induced COX-2 expression by activating PKC- and NFKB-dependent pathways. Stimulation of PKC directly with PMA provoked strong COX-2 expression.

**Conclusions:**

PKC plays a central role in OXT and EGF induced COX-2 expression in human myometrial cells. However, other pathways, notably ERK and NFKB are also involved to an extent which depends on the type of agonist used.

## Background

Preterm birth is defined as birth occurring prior to 37 weeks of gestation. It affects 5-9% of all pregnancies and is associated with more than 50% of long-term morbidity and 75% of neonatal mortality [1,2]. Preterm birth is a worldwide problem affecting developed and developing countries. 15 million babies are born before 37 weeks gestation every year, with one million babies dying from complications [3]. Approximately 7 million children under 12 are affected by sequelae of prematurity, ranging from mild to severe depending on their gestational age at delivery. For example, infants born before 34 weeks gestation have a 40 times higher frequency of cerebral palsy than infants born at term, and the incidence is 90 times higher in infants born before 28 weeks [4,5].

Preterm birth can occur spontaneously (idiopathic) or can be medically induced (iatrogenic) for maternal or fetal indications, such as preeclampsia or intrauterine growth restriction [6]. The underlying mechanisms resulting in spontaneous preterm labour have not been fully elucidated; it is thought that it is either caused by a pathological insult or by an early activation of the mechanism occurring during normal labour at term [1,7].

Inflammation may play an important role in both term and preterm parturition. During labour there is increased synthesis of prostaglandins (PGs) within the uterus via the induction of prostaglandin-endoperoxide synthase (PTGS), also known as cyclooxygenase (COX) [8]. COX is a rate limiting enzyme that is responsible for the conversion of arachidonic acid to prostaglandin endoperoxides, which are further converted into different prostaglandins (PGs) by cell specific terminal synthases [9-11]. Besides relaxation and contraction of the myometrium, PGs also stimulate cervical ripening. This remodelling process involves softening and dilatation of the cervical channel facilitating fetal delivery [12].

There are two isoforms of COX, namely COX-1 and COX-2 (PTGS1, PTGS2). COX-1 is a protein that is constitutively expressed in almost all tissues whereas the expression of COX-2 is highly regulated [11]. Expression of COX-2 in the uterus can be rapidly induced by pro-inflammatory cytokines (e.g. interleukin (IL)1β), growth factors (e.g. epidermal growth factor (EGF)), phorbol esters (e.g. phorbol-12-myristate-13-acetate (PMA)) and by the potent uterotonic hormone oxytocin (OXT) [9,13-16].

OXT is a nanopeptide produced by the hypothalamus and released from the posterior pituitary nerve terminals into the circulation in a pulsatile manner [17]. As labour progresses the frequency of OXT release increases [18,19]. In the myometrium, OXT binds to a specific G protein-coupled receptor (GPCR) linked through Gα_q/11_ to phospholipase Cβ (PLCβ). Activation of PLCβ results in the production of inositol 1, 4, 5-trisphosphate (IP_3_) and diacylglycerol (DAG) [17,20]. IP_3_ releases Ca^2+^ from intracellular stores. In addition, OXT receptor (OXTR) activation increases intracellular Ca^2+^ by stimulating extracellular Ca^2+^ entry through cell membrane channels in human myometrial cells [21]. Increases in intracellular Ca^2+^ are essential to stimulate myometrial contractility; moreover OXTR mediated Ca^2+^ entry stimulates nuclear transcription in human myometrial cells through calcineurin/NFAT (nuclear factor of activated T cells) signalling [22].

DAG is a lipid second messenger that is capable of activating conventional and novel protein kinase C (PKC) isoforms [23]. The phorbol ester, PMA activates PKC by mimicking the function of DAG. Activation of conventional PKC isoforms can however only happen in the presence of both DAG and Ca^2+^[24]. PKC can stimulate different pathways e.g. mitogen-activated protein kinases (MAPK) such as extracellular signal-regulated kinase (ERK), and nuclear factor kappa-light-chain-enhancer of activated B cells (NFKB) both shown to be involved in labour [15,25]. Activation of these pathways can happen under control of PKC or independently of this kinase [26,27]. It has been reported that OXT induces myometrial COX-2 expression and stimulates PG synthesis through activation of ERK and that this process is entirely PKC independent [15,28]. On the other hand, PKC activity has been demonstrated to be important during OXT-mediated myometrial contractions [29]. Recently, we have shown that OXT induces COX-2 mRNA expression in myometrial cells through activation of the calcineurin/NFAT pathway [22].

The growth factor EGF accumulates in the amniotic fluid throughout pregnancy and is present in maternal and fetal blood [30,31]. EGF exerts its function by binding to the EGF receptor (EGFR, the first described member of the proto-oncogene ErbB tyrosine kinase family). Binding of EGF to the EGFR stimulates its tyrosine kinase activity via an allosteric mechanism involving the formation of asymmetric intracellular kinase domains. The phosphorylated EGFR recruits adaptor proteins and activates several major signalling cascades including MAPK, PLCγ, PKC, PI3 kinase-AKT and NFKB [32]. EGF stimulates COX-2 expression in human amnion cells and prostaglandin production in cultured myometrial cells [33,34]. The mechanism of action of EGF has been investigated in many cell types, including amnion cells, and the consensus is that EGFR operates primarily through ERK activation to stimulate COX-2 gene expression, with little involvement of PKC [14,35]; however, the mediators involved in EGFR signalling in myometrial cells have not been described.

The pro-inflammatory cytokine IL1β is a potent stimulator of COX-2 expression in human myometrial cells [36]. During pregnancy the concentration of this cytokine increases in the amniotic fluid [37]. It has been reported that IL1β induces myometrial COX-2 expression via activating PKC and NFKB [27,36].

The aim of this study was to investigate the pathways mediating OXTR and EGFR-induced COX-2 expression in human myometrial cells, and in particular the role of the ERK and PKC pathways. We have also compared the effect of OXT and EGF with the pro-inflammatory cytokine IL1β under the same conditions. The results demonstrate that OXT, EGF and IL1β induced COX-2 expression in myometrial cells is mainly PKC dependent. Our findings contribute towards a better understanding of the mechanisms leading to spontaneous term and preterm labour.

## Results

### Oxytocin stimulates myometrial COX-2 expression via signalling through PKC and ERK

**Figure 1:**
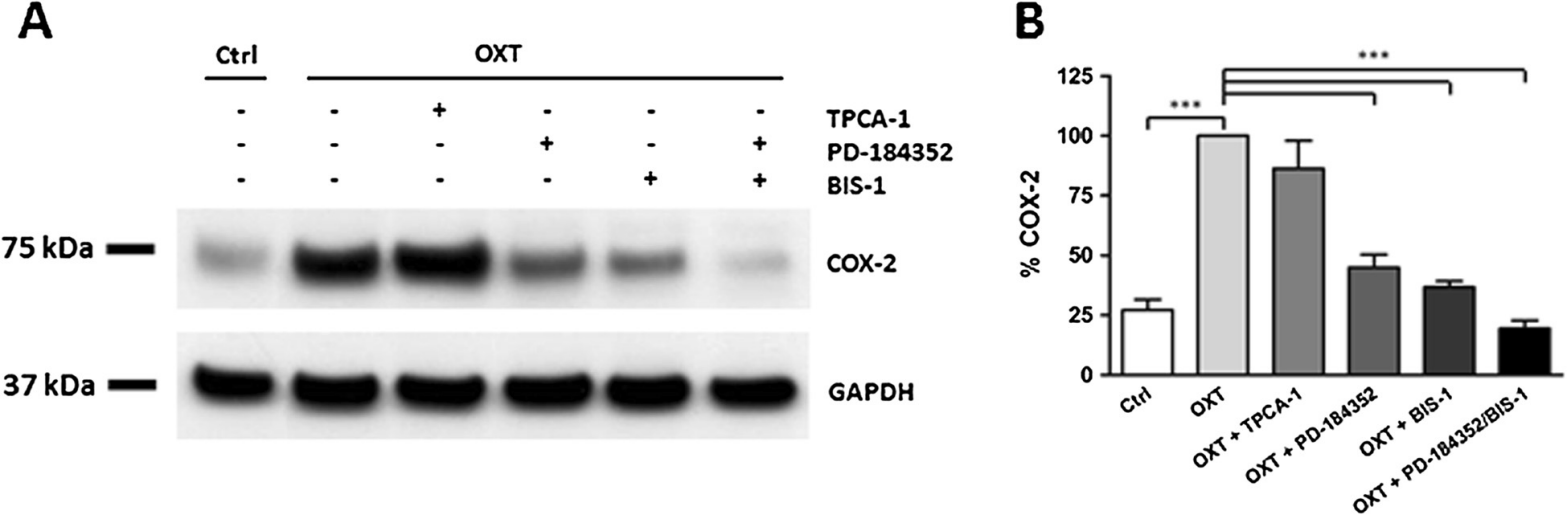
**Mechanism of OXT induced COX-2 expression in human myometrial cells.** Cells were stimulated with 1 μM OXT for 6 hours in the absence and in the presence of different inhibitors. The NFKB pathway was inhibited using TPCA-1 (2 μM). PD-184352 (2 μM) was used to test the ERK pathway. Signalling through PKC was inhibited by means of Bisindolylmaleimide I (BIS1; 5 μM). **(A)** Cell lysates were subjected to immunoblotting for COX-2. Blots were stripped and re-probed for GAPDH. **(B)** COX-2 expression was normalised to GAPDH expression and given as a percentage of the OXT treated value. Data points represent mean ± SEM, n = 5. Statistical significance was determined using one way ANOVA and Dunnett’s *post hoc* test. ***P < 0.001.

Myometrial cells were stimulated with 1 μM OXT for 6 hours in the absence and in the presence of different inhibitors. Signalling through NFKB was inhibited by means of an IKK-2 inhibitor, TPCA-1 (2 μM). The ERK pathway was inhibited using the MEK1/2 inhibitor, PD-184352 (2 μM). Bisindolylmaleimide-I (BIS1; 5 μM) was used to inhibit PKC signalling. After stimulating the cells with OXT they were lysed and subjected to immunoblotting with a COX-2 antibody. Control wells were incubated with inhibitors in the absence of OXT. None of the inhibitors alone had any effect on COX-2 expression (data not shown). As shown in Figure 1, stimulation of myometrial cells with OXT resulted in a fourfold increase (P < 0.001) in COX-2 expression. This increase was significantly inhibited in the presence of PD-184352 (P <0.001) and BIS1 (P <0.001). When both inhibitors were used in combination there was a further decrease in OXT induced COX-2 expression to control levels, but this decrease did not reach statistical significance (Figure 1).

**Figure 2:**
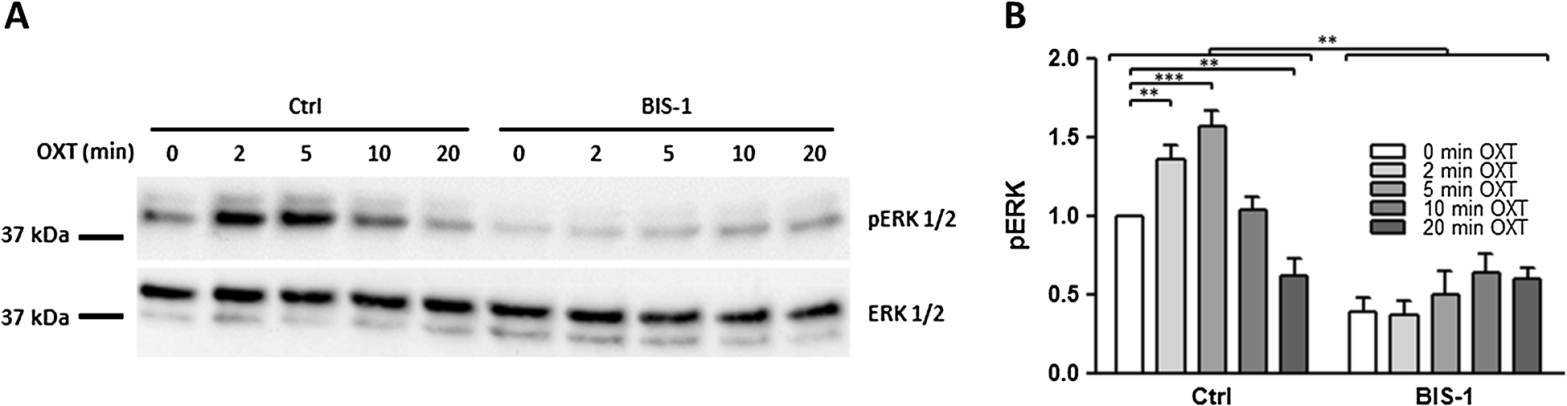
**OXT stimulates ERK phosphorylation in primary human myometrial cells.** Myometrial cells were stimulated with 1 μM OXT for different time points (2-, 5-, 10-, and 20-minutes) in the absence or presence of BIS1 (5 μM). **(A)** Cell lysates were subjected to immunoblotting using a phospho-ERK antibody. Blots were stripped and re-probed for ERK. **(B)** Fold change in ERK phosphorylation normalised to total ERK expression and non-treated values. Data points represent mean ± SEM, n = 3. Statistical significance was determined using two way ANOVA and Bonferroni *post hoc* test. **P < 0.01 and ***P < 0.001.

To demonstrate that the ERK pathway was active in response to OXT, we measured ERK phosphorylation directly. Cells were stimulated with 1 μM OXT for different time points (2-, 5-, 10-, and 20-minutes) with and without pre-treatment with 5 μM BIS1. Lysates were used for immunoblotting with a pERK antibody. Blots were stripped and re-probed for ERK, to which the phosphorylation of ERK was normalised. As shown in Figure 2, ERK phosphorylation increased with OXT stimulation with a maximal response at 5 minutes (P < 0.001). The response to OXT was transient and at 20 minutes pERK levels were below control levels, possibly due to OXT receptor desensitization and phosphatase induction. The PKC inhibitor BIS1 provoked a significant decrease in ERK phosphorylation (P < 0.001).

### EGF stimulates COX-2 expression via signalling through ERK and PKC

**Figure 3:**
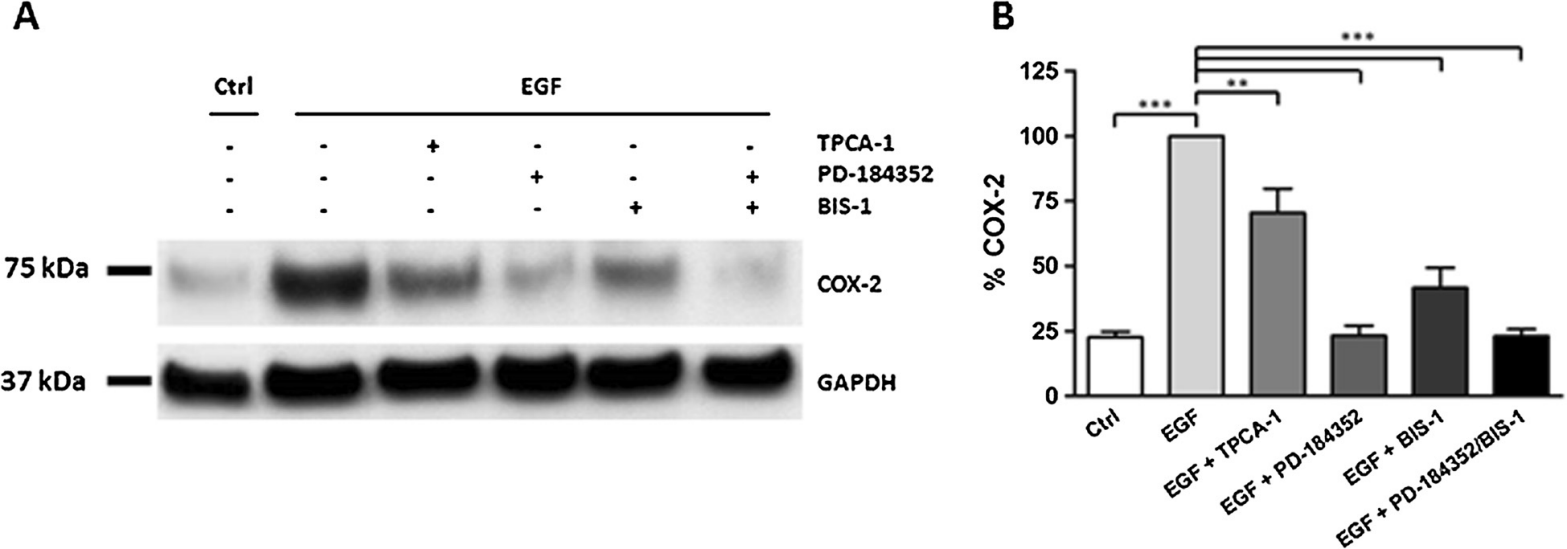
**Mechanisms of EGF induced COX-2 expression in primary human myometrial cells.** Cells were stimulated with 25 ng/ml EGF for 6 hours alone or in the presence of different inhibitors (2 μM TPCA-1, 2 μM PD-184352 and 5 μM BIS1). **(A)** Cell lysates were subjected to immunoblotting using a COX-2 antibody. Blots were stripped and re-probed for GAPDH. **(B)** COX-2 expression normalised to GAPDH expression and given as a percentage of the EGF treated value. Data points represent mean ± SEM, n = 3. Statistical significance was determined by means of one way ANOVA and Dunnett’s post hoc test. ** P < 0.01 and ***P < 0.001.

To investigate EGF induced COX-2 expression, cells were stimulated with 25 ng/ml EGF for 6 hours in the absence and in the presence of different inhibitors (2 μM TPCA-1, 2 μM PD-184352 and 5 μM BIS1). As shown in Figure 3, EGF provoked a significant increase in COX-2 expression (3.9 fold; P < 0.001). This increase was significantly inhibited in the presence of TPCA-1 (P < 0.01), PD-184352 (P < 0.001) and BIS1 (P < 0.001). The inhibitory effect of PD-184352 was the strongest, bringing COX-2 expression down to control levels. TPCA-1 was the weakest inhibitor, with BIS1 having an intermediate effect. The combination of PD-184352 and BIS1 had the same effect as PD-184352 alone (Figure 3).

**Figure 4:**
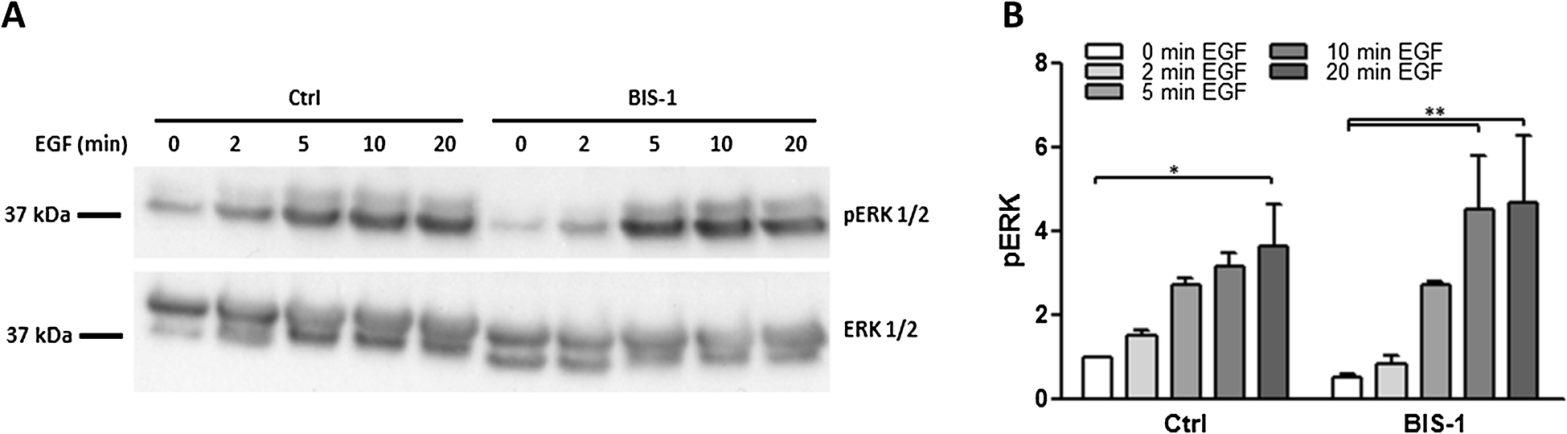
**EGF induces ERK phosphorylation in human myometrial cells.** Cells were stimulated with 25 ng/ml EGF for different time points (2-, 5-, 10-, and 20-min) in the absence or presence of 5 μM BIS1. **(A)** ERK phosphorylation was studied by means of immunoblotting using a phospho-ERK antibody. Blots were stripped and re-probed for ERK. **(B)** Fold change in ERK phosphorylation normalised to total ERK expression and non-treated values. Data points represent mean ± SEM, n = 3. Statistical significance was determined using two way ANOVA and Bonferroni *post hoc* test. * P < 0.05, **P < 0.01.

To study the effect of EGF on ERK activation, cells were stimulated with 25 ng/ml EGF in the absence or presence of 5 μM BIS1 for different time points (2-,5-,10-, and 20-minutes). As depicted in Figure 4, stimulation of myometrial cells with EGF resulted in a marked increase in ERK phosphorylation with a maximal response after a stimulation of 20 min. The PKC inhibitor BIS1 was unable to block this effect (Figure 4) demonstrating its specificity towards PKC in our system.

### IL1β increases COX-2 expression via activating NFKB and PKC

**Figure 5:**
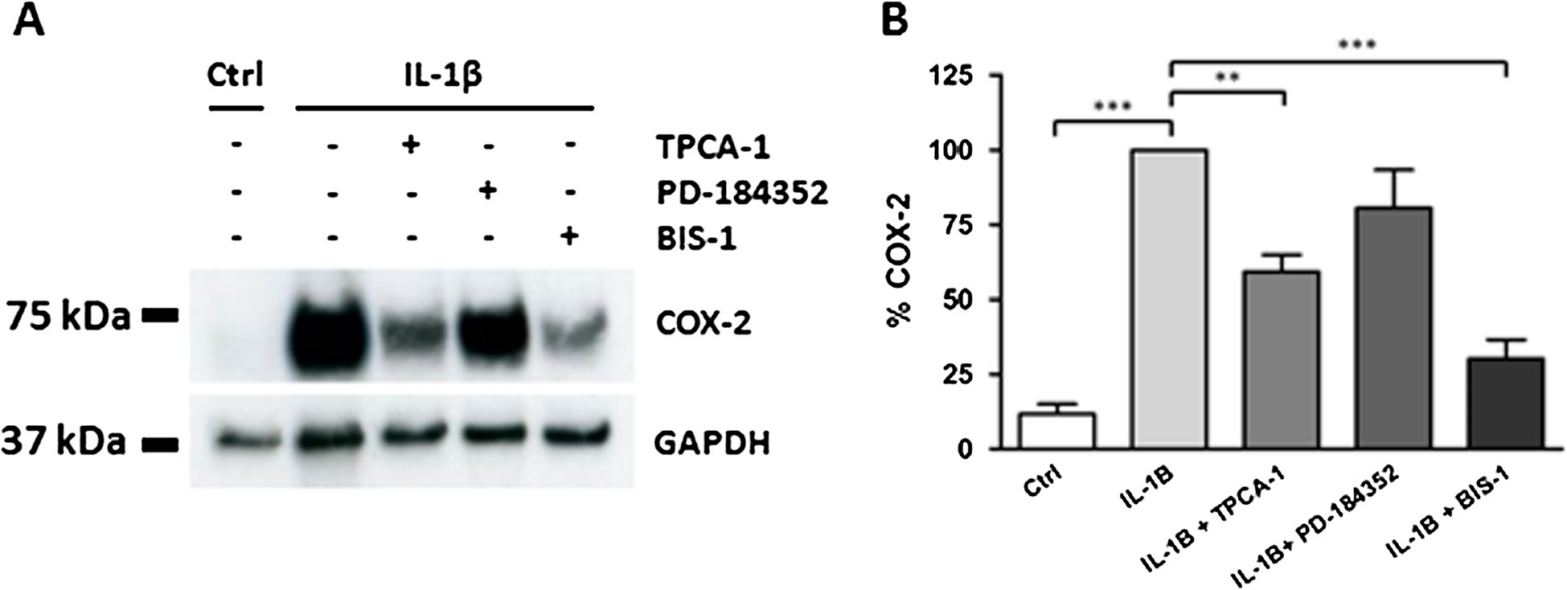
**Mechanisms of IL1β induced COX-2 expression in myometrial cells.** Cells were stimulated with 5 ng/ml IL1β for 6 hours alone or in the presence of different inhibitors (2 μM TPCA-1, 2 μM PD-184352 and 5 μM BIS1). **(A)** Lysed cells were subjected to immunoblotting using a COX-2 antibody. Blots were stripped and re-probed for GAPDH. **(B)** COX-2 expression was normalised to GAPDH expression and given as a percentage of the IL1β treated value. Data points represent mean ± SEM, n = 5. Statistical significance was determined using one way ANOVA and Dunnett’s *post hoc* test. **P < 0.01 and ***P < 0.001.

The pro-inflammatory cytokine IL1β is a potent activator of COX-2 expression. Cells were stimulated for 6 hours with 5 ng/ml IL1β alone or in combination with different inhibitors (TPCA-1 (2 μM), PD-184352 (2 μM) and BIS1 (5 μM)). Cells were lysed and subjected to immunoblotting for COX-2. As expected, IL1β stimulated COX-2 expression in myometrial cells very strongly (8.5 fold; P < 0.001). This effect was significantly inhibited by TPCA-1 (P < 0.01) and BIS1 (P < 0.001) indicating that both NFKB and PKC are involved in IL1β induced COX-2 expression (Figure 5). The ERK pathway on the other hand did not seem to contribute to this effect.

In summary, these results demonstrate that PKC signalling plays an important role in agonist-induced COX-2 expression. PMA activates PKC directly and is a potent activator of COX-2 expression in myometrial cells [27]. We used PMA as a tool to further investigate the involvement of PKC in agonist-induced COX-2 expression in our system.

### PMA induced COX-2 expression: involvement of ERK

**Figure 6:**
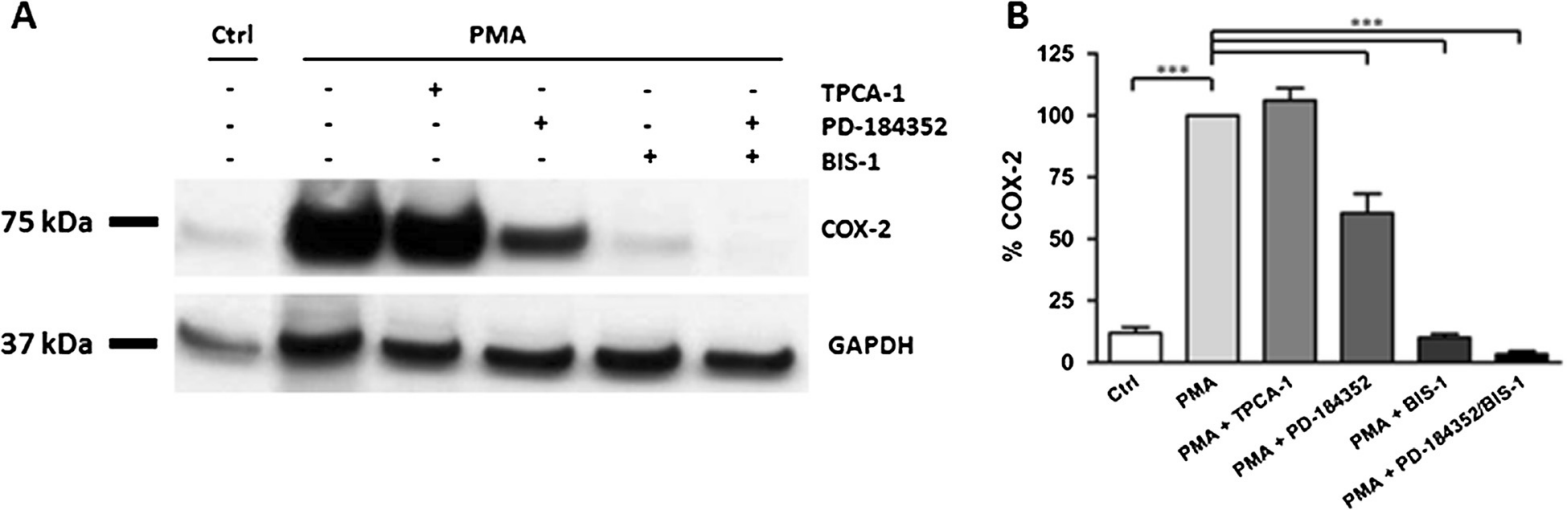
**Mechanisms of PMA induced COX-2 expression in myometrial cells.** Cells were stimulated for 6 hours with the PKC activator, PMA (2 μM) alone or in the presence of different inhibitors (TPCA-1 (2 μM), PD-184352 (2 μM) and BIS1 (5 μM)). **(A)** After lysis the cells were subjected to immunoblotting using a COX-2 antibody. Blots were stripped and re-probed for GAPDH. **(B)** COX-2 expression normalised to GAPDH expression and given as a percentage of the PMA treated value. Data points represent mean ± SEM, n = 5. Statistical significance was determined using one way ANOVA and Dunnett’s *post hoc* test. ***P < 0.001.

Myometrial cells were stimulated with 2 μM PMA for 6 hours alone and in combination with different inhibitors (2 μM TPCA-1, 2 μM PD-184352 and 5 μM BIS1). Effects on COX-2 expression were studied by means of immunoblotting using a COX-2 antibody. As shown in Figure 6, PMA was a potent stimulus for COX-2 expression in human myometrial cells (10.8 fold; P < 0.001). However, the presence of the MEK inhibitor, PD-184352, significantly (P < 0.001) decreased COX-2 expression. The PKC inhibitor, BIS1, completely abolished the effect of PMA on COX-2 expression (P < 0.001).

**Figure 7:**
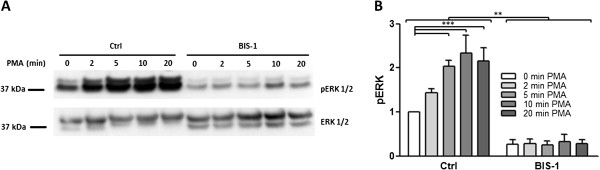
**PMA induces ERK phosphorylation in myometrial cells.** Myometrial cells were stimulated with 2 μM PMA for different time points (2-, 5-, 10-, and 20-minutes) in the presence of 5 μM BIS1.** (A)** Cell lysates were subjected to immunoblotting using a phospho-ERK antibody. Gels were stripped and reprobed for ERK. **(B)** Fold change in ERK phosphorylation relative to non-treated values. Data points represent mean ± SEM, n = 3. Statistical significance was determined using two way ANOVA and Bonferroni *post hoc* test. **P < 0.01 and ***P < 0.001.

To test for ERK phosphorylation, myometrial cells were stimulated with 2 μM PMA for different time points (2-, 5-, 10-, and 20-minutes). Stimulation of myometrial cells with PMA resulted in an increase in ERK phosphorylation with a maximal response after 10 min of stimulation (P < 0.001; Figure 7). Two clear phosphoERK1 and phosphoERK2 bands were detected by the pERK antibody, with preferential phosphorylation of ERK2. The PKC inhibitor BIS1 inhibited this effect (P < 0.001) and also decreased basal levels of ERK phosphorylation.

## Discussion

This study provides the first direct comparison of the effect of OXT and EGF in human myometrial cells and demonstrated that both agonists require PKC activity to stimulate COX-2 expression. Given the importance of COX-2 up-regulation for the onset of labour and the involvement of GPCR and tyrosine kinase receptors in the control of uterine activation [7], we decided to investigate the mechanism by which OXT and EGF increase COX-2 expression in myometrial cells, in particular the role of PKC which has been questioned in previous studies [15].

OXT plays an important role during parturition by stimulating myometrial contractility and promoting the expression of labour-associated genes [22]. Moreover OXT has paracrine interactions with IL1β in human amnion to increase COX-2 expression and stimulate PG production [38]. Increased PG release leads to further paracrine and autocrine effects facilitating uterine activation, for instance PGF2α stimulates OXTR and COX-2 expression in human myometrial cells [39]. Molnar et al. [15] reported that OXT induces COX-2 expression in myometrial cells by activating the ERK pathway and suggested that this is entirely PKC independent. We have confirmed that OXT increases myometrial COX-2 expression via ERK stimulation. However, in contrast to Molnar et al. our study reveals that the stimulatory effect of OXT on COX-2 expression is dependent on PKC activation as shown by the blocking effect of BIS1, a PKC inhibitor. BIS1 had an inhibitory effect on basal levels of ERK phosphorylation at the concentration used, however this is likely to be due to inhibition of constitutive PKC activity in myometrial cells. BIS1 had no effect on EGF-stimulated ERK phosphorylation; hence its inhibitory effect on OXT-induced COX-2 expression is likely to be due to its PKC blocking effect and not due to non-specific inhibition of ERK. It is possible that during pregnancy the myometrium becomes more responsive to PKC signalling [40] and this is reflected in our results.

MEK1/2 and PKC inhibitors were capable of blocking OXT-induced COX-2 expression almost completely when given separately. When they were used in combination, a small additional inhibitory effect was visible. This indicates that both ERK and PKC signalling mediate OXT stimulated COX-2 expression. Moreover, OXT increased ERK phosphorylation and this was significantly inhibited in the presence of BIS1.

The concentration of EGF increases in the amniotic fluid during pregnancy [31]. EGF exerts its function by binding to EGF-receptor dimers and promoting tyrosine phosphorylation of other proteins. It has been shown that EGF activates PKC in human myometrial cells and that it can induce PG production in these cells [33,41]. In human amnion EGF induces COX-2 expression [34,35]. The involvement of EGF in COX-2 expression in human myometrial cells has not been studied before. Our results show that EGF stimulates COX-2 expression in myometrial cells and that this is dependent on the activation of PKC, ERK and NFKB. The inhibitory effect of PD-184352 was the strongest, followed by that of BIS1, with TPCA-1 having only a moderate effect. The addition of BIS1 to PD-184352 produced an inhibitory effect similar to that with PD-184352 alone. This suggests that the ERK pathway is the most important, but there is also a strong PKC component. There is interaction between the two pathways in EGFR and OXT receptor mediated COX-stimulation in bovine endometrial cells [42]. Nevertheless in our human myometrial cells EGF stimulated ERK phosphorylation largely in a PKC independent manner. The role of EGF in stimulating COX-2 expression is worth investigating further, in particular given the capacity of OXT receptors to trans-activate EGFR tyrosine kinase leading to ERK1/2 activation in myometrial cells [43].

IL1β is a potent stimulator of COX-2 expression and exposure of myometrial cells to this agonist resulted in a 9-fold increase in COX-2 expression. It has been shown that IL1β induces COX-2 expression in human myometrial cells by activating the PKC and NFKB pathways [27,36] and we have confirmed this here. Moreover, we have shown that compared to OXT, IL1β is relatively more dependent on the NFKB pathway to stimulate COX-2 expression; however, both agonist rely mainly on PKC signalling.

A potential limitation of this study is that we did not have a positive control for the inhibitor in each individual experiment; however controls are presented throughout the work: for the IKK-2 inhibitor TPCA-1 positive control in Figure 5 and negative control in Figure 6; for the MEK1/2 inhibitor PD-184352 positive control in Figure 3 and negative control in Figure 5; for the PKC inhibitor BIS1 positive control in Figure 6 and negative control in Figure 4. This confirms the consistency of the data.

TPCA-1 is a selective IKK-2 inhibitor that has been extensively characterised in human intrauterine tissues; it inhibits p65 translocation and reduces the expression of genes associated with inflammatory signalling via the NFKB pathway [44]. However IKK-2 also phosphorylates other targets in addition to those involved in NFKB activation, such as beta-catenin and BCL10 which play important roles in cell division and apoptosis. Silva et al. (2010) [44] reported no side effects of TPCA-1 in choriodecidual cells and we did not observe signs of cytotoxicity in our myometrial cell cultures at the doses of TPCA used. PD 184352 is a highly potent non-competitive inhibitor of MEK1 and MEK2; it suppresses the activation of MEK1, rather than blocking its activity. The selectivity of PD 184352 for MEK against a wide range of kinases is excellent [45]. BIS1 is a PKC inhibitor that shows high selectivity for PKC α-, β1-, β2-, γ-, δ-, and ϵ-isozymes in uterine tissues [46]. However, as with other bisindolylmaleimides, the possibility that it also inhibits protein kinases of the RSK and GSK3 families cannot be ruled out [45].

Overall, our results show that PKC plays a central role in agonist-induced COX-2 expression in myometrial cells. When PKC was stimulated directly using PMA there was a large increase in COX-2 expression in the cells. In the presence of PD-184352 a significant decrease in COX-2 expression was observed. BIS1 on the other hand completely inhibited the effect of PMA on COX-2 expression, which shows that PKC can stimulate COX-2 expression independently of ERK. Little or no additive inhibitory effect was observed when the MEK1/2 and PKC inhibitors were used in combination, indicating that the PKC-effect is not entirely ERK mediated.

## Conclusion

**Figure 8:**
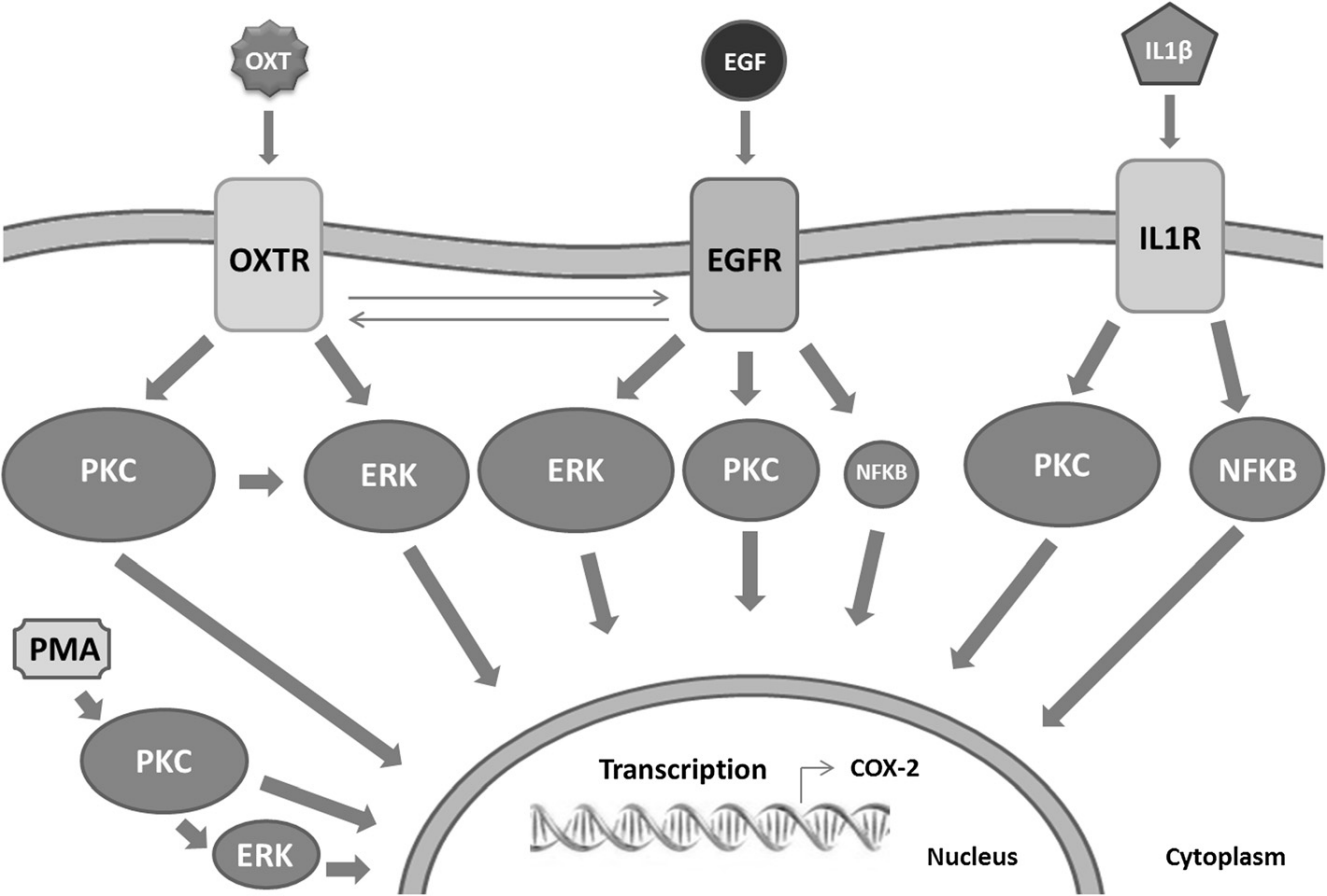
**Schematic diagram.** Underlying signalling pathways activated by OXT, EGF and IL1β to stimulate COX-2 expression in human myometrial cells. The relative importance of each signalling pathway is represented by its size.

In conclusion, there is strong evidence for the involvement of PKC in agonist-induced COX-2 expression in human myometrial cells (Figure 8). This is the most important pathway for both physiological and inflammatory agonists. The effect of PKC appears to lie upstream of ERK but we cannot rule out that the two kinase pathways act independently of each other to convert receptor signalling into COX-2 stimulation. The relative prominence of each pathway in myometrial cells depends on the type of agonist used. These findings contribute to our understanding of myometrial preparation for parturition and respond to the need to investigate myometrial specific mechanisms in order to develop tocolytic drugs with better uterine selectivity. The data will inform further research not only into the prevention of preterm labour, but also into the induction or augmentation of labour where increased expression of COX-2 is important.

## Methods

### Tissue collection

This study was approved by the South West Research Ethics Committee and specimens were collected after obtaining informed written consent. Human myometrial smooth muscle samples were obtained from pregnant women at term (not in labour) undergoing caesarean section at St Michael’s Hospital (Bristol, UK). Caesarean section was performed following maternal request, fetal malposition or previous section. Women with signs of infection were excluded. Myometrial samples were taken from the upper margin of the incision and washed in ice-cold isotonic saline before being transported to the laboratory.

### Myometrial cell culture

Human myometrial tissue was enzymatically dispersed into single cells in serum-free DMEM (Invitrogen, Paisley, UK) containing collagenase type II (300 U/ml; Invitrogen), dispase (0.3 U/ml; Invitrogen), DNase (30 U/ml; Sigma Aldrich, Poole, UK) and elastase (0.09 U/ml; Sigma Aldrich) at 37°C for 4.5 h with shaking. Liberated cells were cultured in T75 flask in DMEM supplemented with 10% (v/v) fetal calf serum (FCS; Invitrogen), 100 U/ml penicillin (Invitrogen) and 100 μg/ml streptomycin (Invitrogen) at 37°C and 5% CO_2_. Cells were seeded at 4 × 10^4^ cells/well in 24-well plates (Greiner Bio-One Ltd, Stonehouse, Gloucestershire, UK) and grown to confluence over 3 days. Cultured primary myometrial cells were used between passages 2–10.

### Agonist stimulation of myometrial cells

Cells were incubated overnight in DMEM containing 2% (v/v) FCS, 100 U/ml penicillin and 100 μg/ml streptomycin. Before treatment, the medium was changed to Krebs solution (4.7 mM KCl, 1.25 mM MgSO_4_, 2.5 mM NaH_2_PO_4_, 1.25 mM CaCl_2_, 130 mM NaCl, 25 mM NaHCO_3_, 11.1 mM glucose, 10 mM Hepes pH 7.6 and 0.1% (w/v) bovine serum albumin (BSA)) and cells were pre-incubated for 30 minutes with inhibitors (TPCA-1 (Sigma Aldrich), 2 μM; PD-184352 (Tocris), 2 μM; Bisindolylmaleimide-I (BIS1; Tocris), 5 μM) followed by the administration of agonists (OXT (Sigma Aldrich), 1 μM; EGF (Merck), 25 ng/ml; IL1β (Cell Guidance Systems, Cambridge, UK), 5 ng/ml; PMA (Sigma Aldrich), 2 μM). Cells were stimulated for 6 hours with a refreshment of medium containing agonists and inhibitors every two hours. After treatment, cells were washed with phosphate-buffered saline (PBS), lysed in boiling sodium dodecyl sulphate polyacrylamide gel electrophoresis (SDS-PAGE) sample buffer (25 mM Tris pH 6.8, 2% (w/v) SDS, 0.002% (w/v) bromophenol blue, 10% (v/v) glycerol and 12.5 mM dithiothreitol) and used for immunoblotting as described below.

For some experiments, cells were pre-incubated with BIS1 for 30 minutes followed by stimulation with agonists for different time points (2-, 5-, 10-, and 20-minutes). Before using for immunoblotting, cells were rinsed in PBS and lysed in boiling SDS-PAGE sample buffer. Cells that were not pre-incubated with inhibitors received 0.1% (v/v) dimethyl sulphoxide (DMSO; vehicle for BIS1) in the presence or absence of agonist.

### Immunoblotting

Samples used for immunoblotting were separated on 8% Bis-Tris gels (357 mM Bis-Tris pH 6.75, 8% (w/v) acrylamide, 0.27% (w/v) bis-acrylamide) in MOPS running buffer (250 mM MOPS, 250 mM Tris, 5 mM EDTA and 0.5% (w/v) SDS). Proteins were transferred by wet transfer to a PVDF membrane (GE Healthcare, Buckinghamshire, UK) at 80 V for 70 minutes in transfer buffer (25 mM Tris, 192 mM glycine and 10% (v/v) methanol). Nonspecific binding was blocked by incubating the membranes in 5% (w/v) BSA in Tris-buffered saline containing 0.1% (v/v) Tween-20 (TBS-T) for 1 hour. Subsequently, membranes were incubating with primary antibodies (COX-2 (Abcam, Cambridge, UK), 1:1000; GAPDH (Abcam), 1:5000; P44/42 MAPK (ERK1/2) (Cell Signaling Technology, Danvers, MA, USA), 1:4000; Phospho-p44/42 MAPK (Cell Signaling Technology), 1:1000) in 5% (w/v) BSA/TBS-T overnight at 4°C. After 6 washes in TBS-T for 5 minutes, membranes were incubated for 1 hour at room temperature with a horseradish peroxidase-conjugated goat anti-rabbit antibody (1:4000; Cell Signaling Technology) in TBS-T. Membranes were washed in TBS-T (6 × 5 minutes) and protein bands were detected with ECL western blot detection reagents (GE Healthcare, Amersham, Buckinghamshire, UK) using chemiluminescence film (GE Healthcare). Films were developed by means of a KODAK X-OMAT 1000 Processor (Kodak, NY, USA). For re-probing, membranes were incubated for 3 × 1 hour in stripping buffer (20 mM glycine and 1% (w/v) SDS, pH 2) followed by incubation in primary antibody. Protein bands were quantified using Quantity One software (Bio-Rad, Hemel Hempstead, UK). Raw data was normalized to the value of the non-stimulated control. COX-2 expression was normalized to the expression of GAPDH and the level of ERK phosphorylation was normalized to the level of total ERK. ERKs are activated by dual phosphorylation of conserved residues within the motif Thr-Glu-Tyr [47]. The phospho-antibody we used recognises ERK1/2 when phosphorylated at Thr202 and Tyr204 (ERK1) or Thr185 and Tyr187 (ERK2) in the activation loop.

### Statistical analysis

Statistical analysis was performed by means of GraphPad Prism (GraphPad Software, La Jolla, CA, USA) using one-way ANOVA followed by the Dunnett *post hoc* test and two-way ANOVA with a Bonferroni *post hoc* test. Values are shown as the mean ± SEM of 3 to 5 replicate experiments using tissue from different donors. Differences were considered statistically significant at P < 0.05. *P < 0.05, **P < 0.01 and ***P < 0.001.

## Abbreviations

BIS: Bisindolylmaleimide; BSA: Bovine serum albumin; COX: Cyclooxygenase; prostaglandin endoperoxide synthase; DAG: Diacylglycerol; DMSO: Dimethyl sulphoxide; EGF: Epidermal growth factor; EGFR: Epidermal growth factor receptor; ERK: Extracellular signal-regulated kinase; GAPDH: Glyceraldehyde 3-phosphate dehydrogenase; GPCR: G protein-coupled receptor; IKK: Inhibitor of kappa light polypeptide gene enhancer in B-cells, kinase; IL1β: Interleukin-1β; IP3: Inositol 1, 4, 5-trisphosphate; MAPK: Mitogen activated protein kinase; MEK: Mitogen activated protein kinase kinase; NFAT: Nuclear factor of activated T cells; NFKB: Nuclear factor kappa-light-chain-enhancer of activated B cells; OXT: Oxytocin; OXTR: Oxytocin receptor; PGs: Prostaglandins; PLCβ: Phospholipase Cβ; PKC: Protein kinase C; PMA: Phorbol-12-myristate-13-acetate; PTGS: Prostaglandin endoperoxide synthase; TPCA: [5-(p-Fluorophenyl)-2-ureido] Thiophene-3-carboxamide.

## Competing interests

The authors declare that they have no competing interests.

## Authors’ contributions

EW carried out the experimental process, analysed the data and drafted the manuscript. CAH supervised the work and participated in the design of the study. CAM edited the manuscript and evaluated the data. ALB designed the work, edited the manuscript and evaluated the data. All authors read and approved the final manuscript.
